# Image and text presentation forms in destination marketing: An eye-tracking analysis and a laboratory experiment

**DOI:** 10.3389/fpsyg.2022.1024991

**Published:** 2022-12-02

**Authors:** Wei Yang, Qiuxia Chen, Xiaoting Huang, Jiaxin Xie, Mei Xie, Jiamin Shi

**Affiliations:** ^1^School of Management, Shandong University, Jinan, China; ^2^Tourism Behavior Laboratory, Shandong University, Jinan, China; ^3^Culture and Tourism Department, Dezhou, China

**Keywords:** destination marketing, tourism advertisement, presentation forms, visit intention, eye tracking

## Abstract

The COVID-19 pandemic has severely impacted the tourism and hospitality industries worldwide. Tourism destination marketing has been an heated focus in tourism and hospitality academia, it is widely believed that it can promote the revival of industries in the post-pandemic era. But there is a lack of research on different graphic presentation forms in tourism advertisements. To bridge the gap in the related literature, this study aims at studying the impact of the image and text presentation forms of the scenic spot’s name in tourism advertisements on tourists’ visit intention to the tourist destination city by combining the theory of constructivism in cognitive psychology, SOR model, and affective-cognitive model to conduct a 2 × 2 between-group experiment. The study found that when the text part contains the scenic spot’s name, the tourism advertisement has a significant impact on tourists’ perceived advertising effectiveness, destination affective image, and visit intention. The results of eye tracking analysis also showed that fixation points are primarily distributed in the text part. Furthermore, this study explored the chain mediating mechanism of perceived advertising effectiveness and destination affective image and discovered that the impact of the text presentation form on the visit intention can be realized through the mediating effect of perceived advertising effectiveness and destination affective image. This study puts forward some suggestions for the tourism advertising and destination marketing of scenic spots with high-familiarity of destination cities with low-familiarity and improving the image of tourist destination cities.

## Introduction

With the rapid development of communication technology, explosive information dissemination has further intensified the competition in the tourism market. Due to the scarcity of attention resources, the era of the “eyeball economy” comes into being. Therefore, tourism advertisement has become the main way to attract people’s attention and carry out tourism marketing activities (Feng et al., 2022; Malvica et al., 2022). When people surf the Internet, use their smartphones to operate on apps, post their status, and share their moods on social media, they will be exposed to various kinds of advertisements (Decré and Cloonan, 2019; Rao and Ko, 2021). At the same time, the eye is the most crucial information receiver of human beings, and most of the information that a person receives from the outside every day is obtained through vision (Zimbardo, 1992). This makes visual marketing different from other sensory marketing such as touch, smell, taste, and hearing. This also makes the visual presentation of information and the resulting visual experience an important factor influencing consumers’ perceptions, decision-making, and even behaviors (Liu et al., 2021).

Scholars in the field of attention economy have noticed that tourism marketing plays an important role in shaping the purchase and visit intention of tourism consumers (Aktan et al., 2022). Moreover, they began to realize the importance of understanding and managing the visual attention of tourism consumers in the era of information explosion (Wang and Sparks, 2016). Some scholars have found that there are significant differences in the impact of watching different destination advertisements on tourism consumers (Zarzosa and Huhmann, 2019). Specifically, it includes the language in advertisements (Selemeneva, 2020), the signs and symbols in advertisements (Lin et al., 2020), the media types of advertisements (Nusair et al., 2021), the characters in advertisements (Zhang et al., 2022), and the aesthetic mystery of advertising design (Shi, 2021). Pan et al. (2013) used eye-tracking techniques to explore people’s visual attention to different hotel search results on search engine pages. It was found that the presence of hotel images on search engine pages attracted more visual attention and people tended to browse hotel information containing images more carefully. Wang and Sparks (2016) compared the visual attention to travel images between Chinese tourists and Australian tourists. The findings suggest that consumers of different races may have different visual processing behaviors. Although there are only a handful of studies on visual attention in tourism (Viglia and Dolnicar, 2020), these studies suggest that tourist viewing behaviors of authentic visual materials used in tourism marketing need to be further studied. In particular, the COVID-19 pandemic has severely impacted the tourism and hospitality industries worldwide (Karabulut et al., 2020), and these industries are in urgent need of revitalization (Jin et al., 2022). At the same time, we noticed that in order to maintain tourists’ interest in destinations during the pandemic and respond to the anticipated rebound of post-pandemic tourism, destination marketing organizations have launched advertising campaigns with different types of appeal (Wut et al., 2021). Therefore, it has become of great value to study the visual behavior rules of tourism marketing (Wen et al., 2020; Medai and Wu, 2022).

This study aims to solve the question: how do different picture and text presentation forms of scenic spot names in destination marketing advertisements affect tourists’ intention to visit the destination cities? The first part of this paper introduces the research objective, significance and relevant literature review. In the second part, the theoretical basis and perspective of this research are explained, and the hypotheses and research framework are put forward. In the third section, the research methodology, process, stimulus materials and data collection process are introduced. In the fourth and fifth part, statistical test, data analysis and eye movement data analysis are carried out, respectively. Finally, the author elaborates the conclusion of the study, discusses with the previous research literature, and explains the limitations of this study and prospects for further research in the future.

## Literature review

In the tourism industry, the majority of consumers (nearly 75%) rely on visual cues to complete their decision-making process (Lee and Gretzel, 2012). Since the 1960s, studies have been conducted to investigate the mechanisms of consumers’ visual cognitive processing of advertisements or packaging with images, especially in terms of visual attention. Many studies have pointed out that both graphic and textual features in advertisements can attract consumers’ visual attention (Percy and Rossiter, 1983, 1997; Singh et al., 2000; Wells et al., 2000; Pieters and Wedel, 2004). Results from advertising research suggest that consumers’ visual attention to advertisements is affected by viewing tasks and viewing time. Consumers tend to spend more time looking at images in advertisements if the task is to remember a brand (Pieters and Wedel, 2007). The Rayner team found that consumers spend more time on the text of an advertisement when they are asked to imagine making a purchase decision rather than being asked to remember the advertisement for later recognition (Rayner et al., 2001, 2008). Pieters and Warlop (1999) found that when consumers are under time pressure, they focus attentively on the image and skip the rest of the advertisement. The Betz team found no impact of the viewing task on viewers’ visual processing behavior (as measured by fixation count) (Betz et al., 2010). In addition to advertisements and packaging, a significant amount of visual attention research has been conducted in the web environment in recent years, including how Internet users view, search, and process information on web pages (Mo et al., 2020). Consumers with negative emotions pay more attention to visual behavior on the screen than consumers with positive emotions (Hwang and Lee, 2022). Kim et al. (2021) team examined how the type and amount of visual and textual information have different effects on consumer responses to experienced products. Most of the above studies on visual processing behavior have used neuroscience techniques such as eye-tracking technology, which has enabled scholars to record viewers’ eye movements and analyze their visual attention. This technology development has provided precise tools for attention research and new perspectives for tourism advertising research (Drèze and Hussherr, 2003; Stewart et al., 2016; Orquin and Holmqvist, 2018).

Apart from the results on visual attention discussed above, scholars have also examined tourists’ perceptions of the effects of destination marketing print advertisements containing images in terms of visual cognitive processing, particularly advertising attitudes and purchase intentions. Empirical studies in this area have examined various contexts, such as print advertisements posted on destination/hotel web pages (Walters et al., 2007; Jun and Holland, 2012) and print advertisements (Laskey et al., 1994). For example, studies have found that the images used in travel agency advertisements positively influence tourists’ attitudes toward the travel agency and their willingness to purchase (Laskey et al., 1994). Moreover, the choice of images is critical because it may lead to different responses from tourists. Pictures presenting tourism products in advertisements are more likely to trigger purchase intention and evoke better imagination about tourism experience in the future than less informative and abstract pictures (Walters et al., 2007). These findings were supported by an analysis of hotel websites, where images showing hotel room features evoked more positive brand attitudes that persuaded consumers to book a room (Jun and Holland, 2012). Print advertising is a common form of advertising in life. It is an intuitively visual carrier that carries the brand and image of a product in a two-dimensional form. Print advertisements includes website ads, advertisements, newspapers, books, brochures, and other promotional materials. The continuous advancement of information technology has resulted in the presentation of print advertising online in many forms, such as ads on destination promotion websites, web banner ads, online pop-up ads, infomercials in portals, and cover ads for a product on e-commerce websites and app applications (Santos et al., 2018). The marketing role of print advertisements as a bridge between merchants and consumers is mainly to present the value of goods (Brito and Pratas, 2015; Leung et al., 2017), to persuade consumers (Kennedy and Guzmán, 2017), to increase consumers’ willingness to buy (Tsang et al., 2004), and to promote the communication of the destination image (Zhou, 1997; Byun and Jang, 2018). Tourism print advertisement serves as a promotional message that is transmitted to the cognitive and affective systems through visual processing, thus affecting destination selection and visit intention of tourists (Simola et al., 2013; John and De'Villiers, 2020). A study by Wang et al. (2022) using an eye-tracking analysis found that marketing advertisements that combined natural movement scenes with health promotion-focused messages were more likely to be noticed and selected by older consumers. Li et al. (2022) found that celebrity endorsement of destinations significantly increased tourists’ visual attention to tourism advertisements, positive destination attitudes, and visit intention.

Eye tracking studies are commonly conducted in a laboratory due to the accuracy and stability requirements of the equipment. Participants are required to view visually stimulating material on a computer screen while their eye movements are recorded with an eye tracker. Researchers typically use a variety of measurements to statistically analyze eye movements, such as fixation duration, fixation position, fixation counts, saccade counts, and sweeping gaze (Duchowski, 2002). What’s more, several types of image presentations have been used to illustrate visual attention patterns, such as gaze maps (representing the point at which the participant’s eyes focus), areas of interest (a high rate of gaze in an area indicates that the participant is interested in that area), heat maps (color-coded visualizations showing where the participant is gazing), and scan paths (the order in which the participant’s eyes focus). While most previous studies have used artificial materials such as text, pictures, and researcher-designed advertisements as visual stimuli, more and more scholars are interested in using natural materials such as web pages, advertisements, and commercials that are not modified to explore visual processing behavior in real world.

Some scholars pointed out that when tourists perceive a large amount of information (e.g., tourism advertising), their sensitivity will decrease. As a result, the effectiveness of tourism advertising stimuli will decrease correspondingly (Ha and Mccann, 2008; Djamasbi et al., 2016). Therefore, it is pivotal to understand tourists’ preferences and choices through an in-depth assessment of potential mechanisms related to tourists’ perceptions of tourism advertisement attributes, such as formal content (e.g., text and images) and orientation (e.g., position of presentation and form of distribution). While previous studies have identified the impact of tourism advertising on tourist behavior (Campelo et al., 2011; Simola et al., 2013; Scott et al., 2017; Byun and Jang, 2018; Kong et al., 2019; Wang et al., 2019), many of these prior studies have yet to answer why some advertisements are effective.

Based on the related literature review, scholars have found that most of the studies on tourism advertisements focus on content design, spokesperson, presentation of scenic pictures, or introduction of cultural scenarios. However, there is a lack of research on the impact of the relationship between the textual and graphic elements of tourism advertisements and the form of position on the tourists’ visit intention. Tourism advertisements generally consist of an image part and a text part. The actual photography of the most well-known scenic spots in tourist destinations is an important element in the content design of many tourism advertisements. The scenic spot’s names appearing in the advertisement can often become the highlight of the destination city promotion. To be specific, in terms of the design of tourism advertisements, the names of representative scenic spots in these destination cities can be presented in the image part of tourism advertisements in the form of images. For example, the names of scenic spots are naturally embedded as part of the landscape of tourism advertisements, which can be in the form of stone monuments, plaques, words on mountains, etc. Another form is that the names of scenic spots can be presented in the text part of tourism advertisements in the form of words, such as in the promotional slogans, destination profiles, etc. To bridge the gap in the related literature, According to the representative scenic spots in tourism destination cities, this study divides the presentation form of the scenic spot’s name in tourism advertisements into two dimensions: image presentation form and text presentation form. This study examined the impact of different of the image and text presentation forms of the scenic spot’s name in tourism advertisements on tourists’ visit intention to the tourist destination city by combining the theory of constructivism in cognitive psychology, SOR model, and affective-cognitive model to conduct a 2 × 2 between-group experiment.

## Hypotheses development and theoretical foundation

### Perceived advertising effectiveness and destination affective image

Destination affective image is a concept that considers the evaluation and judgment of destination by individuals from the affective dimension, and has received extensive attention in the literature (e.g., Woosnam et al., 2020; Najar and Rather, 2022; Yang et al., 2022a). Destination affective image is related to the individual’s emotional response and feeling towards destination (Wang and Hsu, 2010). An individual’s impression of a place can lead to behavioral intentions, for example, the possibility of travel intention and post-visit behaviors (Wu and Liang, 2020; Marques et al., 2021). Destination affective image will affect the appraisal, decision and expectation of tourism consumers, etc (Akgün et al., 2020; Perpiña et al., 2021; Wu and Lai, 2021). Therefore, destination marketers are very interested in the process of destination affective image formation. Destination affective image is initially formed through multiple interactions of individuals with various stimuli, including overt advertising, covert public relations, autonomous consumption of media, and oral stories (Gartner, 1994; Norton, 1996). The opportunity to influence the early stages of an individual’s destination affective image is an important consideration when forming an affective relationship with a possible future destination.

Zeitham (l988) proposed the theory of perceived value and perceived value is regarded as the overall evaluation of products or service made by consumers based on a comparison of benefits and costs during the entire purchase and use process. Dodds et al. (1991) proposed that perceived value is the ratio of the customer’s perceived benefits to the perceived losses in the study of consumers’ product value measurement. Anderson and Narus (1998) argued that perceived value is consumers’ comprehensive perception and evaluation of the economic, technical, and social value of a product or service. Balasubramanian et al. (2006) argued that the amount of information presented in an advertisement and the audience’s emotions while watching the advertisement would affect its perceived effectiveness. Zhao et al. (2022) showed that the spatial layout form of tourism destination advertisements has an impact on the perceived effectiveness of advertisements and tourists visual attention. Other studies have found that consumers’ perceived effectiveness of destination promotional advertisements may shape their destination affective image (Hultman et al., 2017; Xu, 2022). The tourism advertisement is a particularly common marketing tool in tourism and is an effective medium for destination managers to establish the destination image and stimulate consumers’ motivation to travel, purchase, and visit (Wang et al., 2016; Lv and McCabe, 2020; Amatulli et al., 2021). Based on the above analysis, hypotheses are proposed in the following:

*H1*: Each dimension of presentation form of the scenic spot's name in tourism advertisements has a significant impact on tourists' perceived advertising effectiveness.

*H1a*: The image presentation form of the scenic spot's name in tourism advertisements has a significant impact on tourists' perceived advertising effectiveness.

*H1b*: The text presentation of the scenic spot's name in tourism advertisements has a significant impact on tourists' perceived advertising effectiveness.

*H2*: Each dimension of presentation form of the scenic spot's name in tourism advertisements has a significant impact on the tourists' destination affective image.

*H2a*: The image presentation form of the scenic spot's name in tourism advertisements has a significant impact on the tourists' destination affective image.

*H2b*: The text presentation form of the scenic spot's name in tourism advertisements has a significant impact on the tourists' destination affective image.

*H3*: Perceived advertising effectiveness of tourism advertisements has a significant impact on the tourists' destination affective image.

*H3a*: Based on the image presentation form, the perceived advertising effectiveness of tourism advertisements has a significant impact on the tourists' destination affective image.

*H3b*: Based on the text presentation form, the perceived advertising effectiveness of tourism advertisements has a significant impact on the tourists' destination affective image.

### Perceived advertising effectiveness and visit intention

Perceived advertising effectiveness can assess whether an advertisement will have a positive impact on consumers. In tourism advertising communication contexts, it has been used to assess the perceived advertising effectiveness of tourists’ perceptions of tourism photos with different texts about the tourist attractions (Li et al., 2016). Tourists’ perceptions of tourism advertisements will affect their attitudes, thus, contributing to tourists’ willingness to purchase (Walters et al., 2007). Empirical studies in this research field have examined various settings such as print advertisements (Salameh et al., 2022) and destination promotion advertisements (Walters et al., 2007; Jun and Holland, 2012). A recent study adopting a convolutional neural network approach also proved that the perceived effectiveness of tourism advertisements images contributes well to visit intention (Malvica et al., 2022). An study, respectively, investigated the impact of visual stimulus information in advertisements and tactile stimulus cues in advertisements on consumers’ purchase intention (Decré and Cloonan, 2019). Rao and Ko (2021) studied the influence of WeChat mini program advertising perception effect on consumer purchasing behavior based on WeChat platform (the most popular mobile social media app in mainland China). In this study, the perceived advertising effectiveness of tourism advertising primarily aims at measuring the level of visitor’s interest and information usefulness, and it is believed that the attitudes generated by tourists viewing tourism advertisements will affect the visit intention to the destination, so the following proposes hypotheses:

*H4*: Perceived advertising effectiveness has a significant impact on visit intention.

*H4a*: Perceived advertising effectiveness has a significant impact on visit intention when presented in image form.

*H4b*: Perceived advertising effectiveness has a significant impact on visit intention when presented in text form.

### Destination affective image and visit intention

Jenkins (1999) proposed that the tourism destination image is influenced by individual internal factors and external stimuli, based on which he proposed that the formation of the tourism destination image is a static process. Baloglu and McCleary (1999) proposed a tourism destination image formation model based on Jenkins’ research, including sensory image (personal factors), evoked image (stimulus factors), and the overall image. He believed that sensory image and evoked image jointly form the overall image. However, other scholars argued that tourism destination formation is a dynamic process. Gunn (1988) clearly proposed that tourism destination image can be divided into original image and induced image. Based on that, Fakeye and Crompton (1992) classify tourism destination images into sensory images, evoked images, and composite images. Baloglu and McCleary (1999) deemed the theory of tourism destination image includes both personal and stimulus factors. Through their research, they concluded that the information sources in the stimulus materials will affect individuals’ cognitive image, and the psychological and social factors in the personal factors will affect individuals’ perception of the destination affective image, which has a corresponding impact on the destination affective image. Destination image influences tourists’ and potential tourists’ cognitive image and affective image of tourist destinations, which indirectly influences tourists’ and potential tourists’ travel decisions. Qu et al. (2022) found that a good destination affective image positively and significantly influenced tourists’ intention to visit Hangzhou. The destination affective image affects tourists’ tourism experiences, and tourists’ evaluations of their experiences, in turn, affect post-travel behaviors such as revisit intentions, recommendation behaviors, and positive word-of-mouth (Papadimitriou et al., 2015; Tosun et al., 2015; Afshardoost and Eshaghi, 2020; Huang et al., 2020; Cham et al., 2022). Based on the above analysis, the following hypotheses are proposed:

*H5*: Destination affective image has a significant impact on visit intention.

*H5a*: Based on the image presentation form, the destination affective image has a significant impact on visit intention.

*H5b*: Based on the text presentation form, the destination affective image has a significant impact on visit intention.

### Cognitive theory and SOR framework

According to cognitive theory, the visual process is a perceptual conclusion made by the viewer’s active brain activity. After watching the stimulus materials, the viewer will utilize all cognitive psychology, such as sensation, perception, association, imagination, emotion, understanding, memory, representation, and others so as to grasp the characteristics and connotation of the stimulus. The process of watching is not a passive reflection of the objective world, but an active process to actively construct. Through the process of construction, the participant not only reflects the objective world but also “constructs” or “creates” the objective world (Zhu et al., 2017; Khoi et al., 2021).

The SOR framework, composed of stimulus, organism, and response, was proposed by Mehrabian and Russell (1974) as a theoretical model for studying the role of individual-related stimuli on an individual’s cognitive or psychological response and subsequent behaviors, such as convergence or avoidance. SOR model has been widely used in the field of tourism consumer research (Prodanova et al., 2020; Jiang, 2022; Rahman and Nguyen-Viet, 2022; Sharma et al., 2022; Yang et al., 2022b). Sharma et al. (2022) applied SOR model framework to the impact of SMS advertising on consumers’ perception of advertising value and advertising attitude. Dogru et al. (2021) compared the impact of dynamic picture ads and static picture ads on consumers’ perception of advertising value. Yadav et al. (2021) used SOR model framework to investigate the impact of electronic word-of-mouth (eWOM) on consumer destination preferences and intentions. Yang et al. (2022a,b) carried out an experiment designed between 2×2 subjects, and used SOR model framework to investigate the VR advertising perception, emotion and destination visit intention of tourists by destination VR marketing advertising. Li et al. (2022) used SOR model framework to investigate the impact of video advertising characteristics on consumer sentiment and behavior.

The present study used the SOR framework for the operationalization of the construct. The SOR framework describes that there exist several environmental stimuli that are responsible for initiating the internal processes such as thoughts, emotions and perception (Bagozzi, 1986), which further influences the final reactions or responses (Chopdar and Balakrishnan, 2020). The organism requires internal cognitive and emotional processes that are seen in the SOR framework as intermediate states between stimulus and response (Fang et al., 2017).

So, it is inferred that the consumers’behavior are not influenced by the environmental stimuli directly, instead it follows an indirect route with the help of a mediation mechanism, in which the stimuli lead to internal processes and that finally lead to the final behavioral outcomes by the consumers. Therefore, the SOR framework is a well-suited and logical choice for our study following the nature and ingredients of this study. Based on the SOR logical framework, in this study, the stimulus (S) refers to the graphic information of tourism advertisements; the organism (O) is the tourist’s perceived advertising effectiveness and destination affective image, and the response (R) is the tourist’s visit intention. This study tries to manipulate the graphic presentation forms of tourism advertisements to induce changes in the tourists’ perceived advertising effectiveness and destination affective images, thus influencing the tourists’ visit intention.

### Affective-cognitive model

The affective-cognitive model of individual’s decision-making is a dual process theory proposed by Shiv and Fedorikhin (1999), which indicates that consumers develop two processing procedures, affective-oriented and cognitive-oriented, in their decision-making activities, illustrating the conflict between the mind and the heart in the consumer’s decision-making process. The affective-cognitive decision model reveals the role of emotion and cognition in different situations in the decision process (Li and Huang, 2020). Consumers choose the method of processing information through the availability of information resources, which reflects the relationship between external stimuli and information processing configurations (Yan and Bai, 2004). If decision information resources are sufficient, affective-oriented processing will be performed first to quickly assess the importance of emotions, and then cognitive orientation will be activated to generate solid support reasons for making decisions (Ahmad and Guzmán, 2021). On the contrary, if information resources are insufficient, affective-oriented processing will be limited, which in turn will affect cognitive-oriented processing and participants’ decision-making (Keer et al., 2010). Such a mechanism can help people to cope with conflicting information and avoid information overload (Carrasco, 2011).

The affective-cognitive model refines the process of the influence of information processing resources on consumers’ ability to process advertising information in different contextual stimulating situations. In the context of watching an advertisement, the way consumers process advertising information depends on the number of information resources consumers receive from the advertisement. When some consumers view advertisements, the lack of sufficient motivation to process information resources will result in insufficient information processing resources, thus causing consumers to trigger emotion-oriented processing while viewing advertisements. That’s to say, decision-making is affected by both affective-oriented and cognitive-oriented dual processing processes (Boo and Busser, 2018; Ceylan and Çizel, 2018; Kim et al., 2020; Yilmaz and Yilmaz, 2020). The affective-cognitive model is used as the basis of the model which posits that e-servicescape of the online travel and tourism websites, through the intervening effects of experiential-affective and functional-cognitive routes, influence the outcomes of online travel and tourism behaviors in terms of e-loyalty (Sreejesh and Abhilash, 2017). Therefore, this study deduces that both the perceived advertising effectiveness that is based on cognitive-oriented processing and the destination affective image that is based on affective-oriented processing as mediating variables mediate consumers’ processing of advertising content information to generate visit intention to tourist destinations. Based on the above theoretical and mechanistic analysis, this paper proposes the following hypotheses:

*H6*: The graphic presentation form of tourism advertisements has a significant impact on visit intention.

*H6a*: The image presentation form of tourism advertisements has a significant impact on visit intention.

*H6b*: The text presentation form of tourism advertisements has a significant impact on visit intention.

*H7*: Perceived advertising effectiveness mediates the impact of each dimension of the presentation form of tourism advertisements on tourists' visit intention.

*H7a*: Perceived advertising effectiveness mediates the impact of the image presentation form of tourism advertisement on tourists' visit intention.

*H7b*: Perceived advertising effectiveness mediates the impact of the text presentation form of tourism advertisements on tourists' visit intention.

*H8*: Destination affective image mediates the impact of each dimension of the presentation form of tourism advertisements sing on tourists' visit intention.

*H8a*: Destination affective image mediates the impact of the image presentation form of tourism advertisements on tourists' visit intention.

*H8b*: Destination affective image mediates the impact of the text presentation form of tourism advertisements on tourists' visit intention.

*H9*: The impact of each dimension of presentation forms of tourism advertisements on tourists' visit intention can be realized by the chain mediating role of perceived advertising effectiveness and destination affective image.

*H9a*: The impact of the image presentation form of tourism advertisements on tourists' visit intention can be realized through the chain mediating role of perceived advertisements effectiveness and destination affective image.

*H9b*: The impact of the text presentation form of tourism advertisements on tourists' visit intention can be realized through the chain mediating role of the perceived advertising effectiveness and destination affective image.

## Research design and data collection

### Research design

This research conducted a between-group experiment with 2 (image containing the scenic spot’s name and image without containing the scenic spot’s name) × 2 (text containing the scenic spot’s name and text without containing the scenic spot’s name) to examine the proposed hypotheses.

#### Tourist destination selection

In order to study the impact of different forms of tourism advertisements on visitor intention to travel to scenic spots with high familiarity in cities with low familiarity, a typical city-landscape combination with low city familiarity and high landscape familiarity should be selected. The selected city is used as the subject for tourism advertisement design and the scenic spot’s elements are controlled for experimental design.

In order to select a combination that is more in line with experimental requirements, this study designed a questionnaire for tourism destination familiarity. The concept of destination familiarity was introduced into tourism research by scholar Cohen in 1972, and many scholars later conducted more in-depth studies on destination familiarity pairs, but so far there is still no controversy about the measurement dimensions of destination familiarity. Until 2001, the three-dimensional view highlighted by Baloglu (2001) was generally accepted. According to Baloglu (2001), the dimension of destination familiarity is not merely information familiarity or experience familiarity, but a combination of the two. This paper also draws on Li′s study to divide the dimensions of destination familiarity into three dimensions: experience familiarity, information familiarity, and proximity familiarity. The experience familiarity is measured by the number of visits. The information familiarity is measured by the number of ways to get information and whether visitors know that the destination is in the city. The proximity familiarity was measured by with or without friends and relatives in the city. The final questionnaire consisted of 11 questions (Supplementary Appendix Table S1).

In order to ensure a high familiarity with the scenic spots, the tourist destinations selected for this study are all within the scope of China’s World Heritage, World Natural Heritage, or World Cultural and Natural Heritage. For the sake of accuracy, combinations of destination cities and scenic spots being highly similar were excluded, such as Huangshan City, and Huangshan Scenic Spot. In addition, instead of selecting multiple combinations of the same province, a combination of one city and one scenic spot was selected for each province to make the alternative more widely distributed. Finally, we select 10 combinations (shown in Supplementary Appendix Table S2).

To ensure that the visit to the place falls under the category of tourist activities, this study censored the data of locals and residents. The final number of valid questionnaires in each of the 10 groups was above 150, adding up to 1,500. By drawing on the research of Baloglu (2001), this study would calculate the familiarity score. Finally, the combination of Jiujiang-Lushan was selected as Jiujiang has low familiarity and Lushan has high familiarity, and the difference between the scores was the largest, which is in line with the purpose of this study.

#### Stimulus materials

The tourism advertisement design chose Jiujiang City-Mount Lu as the combination. Four advertisements of Jiujiang city were designed by Adobe Photohop software as the experimental stimulus material. Besides the fundamental elements, the image part and the text part have an alternative element, which is a stone monument image engraved with the characters “Lushan” in red in the image part. The alternative text part is “To know the true face of Lushan, please come to Jiujiang, Jiangxi.” With fundamental elements remaining unchanged, the experiment is controlled by adding or not adding alternative elements to the advertisements.

The fundamental elements of the color, location font, etc. remain unchanged while the image and text part will change. That’s to say, the image part will be with or without the name of the landscape, namely a Mount Lushan stone inscription. The text part will add or will not add the sentence ‘to know the true face of Mount Lushan, please come to Jiangxi Jiujiang’. The advertisement image is divided into two kinds: the image part contains the scenic spot’s name and the image part does not contain the scenic spot’s name. What’s more, advertisement text is also divided into two kinds: the text part contains the scenic spot’s name and the text part does not contain the scenic spot’s name. The specific design is as follows:

Group A: The image part contains the scenic spot’s name, but the text part does not contain the scenic spot’s name.

Group B: The image part does not contain the scenic spot’s name, and the text part contains the scenic spot’s name.

Group C: Both image and text parts contain the scenic spot’s name.

Group D: Neither the image nor text parts contain the scenic spot’s name.

#### Participants

A total of 120 participants were recruited for this experiment, aged 18–35 years (M_age_ = 23.5), and all participants had not been to Jiujiang City before this experiment. Affected by the covid-19, strict policies of contact restriction had been launched in many cities. Therefore, many people were quarantined in their homes because of the severe pandemic explosion. College students were quarantined on their campuses so we chose college students as participants, including MBA students. The distribution of the sample data in this study includes: 16 (15.1%) are male; 72 (38%) are undergraduate students and 34 (32%) are postgraduate. Thus, our participants had good cognitive and comprehensive abilities. The vast majority of the participants had no experience as participants in the experimental study, and there was little chance of guessing the purpose of the experiment due to the rich participant experience. Before starting the experiment, the research assistants were required to check whether the participants were in a good mental condition to participate in the experiment, and this was done to avoid the participant effect. The experiment required participants to have bare or corrected visual acuity of 1.0 and above and no astigmatism. After deleting invalid data, the final valid data was 106, and the effective rate was 88%. We randomly assigned all participants to groups A, B, C, and D. We prepared a souvenir worth 20 RMB for each participant as a reward for their participation in this experiment.

#### Experimental environment

In this study, the Tourism Behavior Laboratory (TBL) of one University in Shandong, China was selected as the experimental site. The Travel Behavior Laboratory has a microclimate virtual simulation laboratory, virtual reality experimental devices, eye-trackers, and so on. The eye-tracker can test any stimulus material displayed on the screen, including images, videos, documents, web pages, questionnaires, experimental procedures, games, etc., and collect eye movement data. The laboratory environment is definitely suitable for conducting eye-movement experiments. Only one participant can enter the laboratory at a time, providing a good environment for participants, and setting the appropriate light and temperature to ensure that participants can complete their experimental tasks in a comfortable and flexible environment (Espigares-Jurado et al., 2020).

#### Experimental equipment

This experiment used the Eyeso Ec60 telemetric eye-tracking device. The EyeSo Ec60 telemetric eye-tracking device is a professional eye-movement experiment platform with complete functionality and high value. It can be used in conjunction with EyeSo Studio experimental design software to enable independent experiments. The EyeSo Studio does not need to be worn by participants, allowing the experiment to be performed in a natural state (Wästlund et al., 2015).

Eyeso studio is an advanced comprehensive eye-movement data analysis software that fully integrates experimental design and eye-movement data recording functions, with various eye-movement data analysis modules such as fixation trajectory map, heat map, and AOI map. It is suitable for experimental environments in various research fields based on screen-based experimental design and presentation.

### Experiment procedures

#### Pre-experiment

Since some participants claimed that they did not know the traditional Chinese characters for “Lushan,” the traditional Chinese characters for “Lushan” were changed to simplified Chinese through Adobe Photoshop software to avoid possible experimental errors. Through the pre-experiment, it was found that setting the viewing time to 30 s might make the participants feel anxious. After multiple measurements, 60 s was finally considered more appropriate. The experiment will use a diagram to show the calibration process to the participants and explain the experimental procedure with a more vivid and easy-to-understand expression. Research assistants will remind the participants many times during the pre-explanation and the interval of each session. In addition, some expressions in the questionnaire were modified based on the participants’ feedback. In order to test the feasibility of the experimental design, this study conducted a pre-experiment before the formal experiment. 12 participants were randomly assigned to groups A, B, C, and D. The purpose of this pre-experiment was to refine the design of the stimulus materials, the experimental procedure, and the questionnaires. The whole experimental procedure lasted approximately 10 min. After the pre-experiment, participants’ problems with the stimulus materials, the experimental procedure, and the questionnaire process would be recorded and modified.

#### Experiment steps

First, when the participants arrived at the laboratory and took a short break, the research assistant introduced the eye-movement experiment procedure and the purpose of the experiment to the participants. Participants signed the informed consent form and filled in the basic information (e.g., whether they had been to the target city before). According to Germine et al. (2012), in terms of procedural control, this study concealed the purpose of the study and the names of the variables when designing the questionnaire beforehand to avoid participants to speculate the purpose of the study. Only one participant was allowed to enter the laboratory at a time to make sure that the experiment was not disturbed.

Second, eye-tracking device calibration was performed. The experiment consisted of two main important parts, the first was to perform the instrument calibration and the second was to view the stimulus material. To ensure accurate data collection, the participant had to keep his or her head still throughout the experiment, for approximately 2 min. On the premise that the success of an eye-tracking experiment needs to ensure calibration accurately, each participant had to experience the calibration process. First of all, the participant sat in front of the computer, ensuring a comfortable sitting position and a distance of approximately 60 cm from the eye-tracking device. The manuscript illustration was presented in front of participants so they knew what was going to happen during the calibration process. The research assistant would remind participants of looking at the six dots on the screen and keeping their heads still and staring at each blue dot for about 2–3 s. After the calibration, the screen will return to the pre-calibration page, and the research assistant would check the calibration results. After successful calibration, the screen displayed a tourism advertisement, the viewing time was 60 s, and the page exited automatically after viewing. We set 60s as the viewing time because of two reasons: first, some pre-interview studies had been conducted before the formal experiment, and 60s were considered the optimal exposure time; second, according to the advertising literature, an appropriate fixation exposure time could avoid participants from completing viewing the material in too short a time, resulting in eye-movement that could not be adequately analyzed (Wang and Sparks, 2016; Bergkvist, 2017; Wang and Hung, 2019).

Third, participants filled in the questionnaire. After completing the eye-tracking experiment, the participants completed the questionnaire according to the real feelings they got from watching the travel advertisement. At the end of the experiment, the participants would receive souvenirs and leave the laboratory.

#### Ethical issue

Participants had be informed of the experimental setting, equipment, and procedures before being recruited. The research assistant assured participants that the experiment was conducted anonymously and eye movement data collected would only be used for academic purpose. All participants received the informed consent form and signed it voluntarily. At the end of the experiment, they had received rewards and known the true purpose of this experiment. All participants completed the experiment in a relaxed environment and could stop at any time if they felt uncomfortable. There were no negative impacts on their physical or mental health from this experiment.

### Data collection

#### Eye movement data

The experimental data were processed by using EyeSo Studio software, and a total of 106 samples were obtained. The data of fixation positions, fixation duration, average fixation duration, and fixation counts of participants were collected. The fixation position refers to the coordinates of the fixation point in the screen; the fixation duration refers to the length of time a participant gazes at the specific area of the picture (Rayner et al., 2008; Scott et al., 2017); the average fixation duration refers to the average length of time a participant gazes at the specific area of the picture; the fixation counts refers to the frequency that a participant stays in an area as a reflection of the participant’s AOI of the specific content inside the picture (Hutton and Nolte, 2011).

#### Questionnaire data

The questionnaire consisted of 11 items, used to measure the participants’ perceived advertising effectiveness, destination affective image, and visit intention. A 5-point Likert scale (‘1’ = strongly disagree and ‘5’ = strongly agree) was adopted.

## Data analysis

### Reliability and validity

#### Reliability test

The number of valid sample in this study was 106. We use SPSS to test Cronbach’s alpha of each construct to measure the internal consistency of questionnaires. The overall reliability of the questionnaire on perceived advertising effectiveness, destination affective image, and visit intention was 0.902, which was greater than 0.7, indicating that the scale was very reliable.

#### Validity test

The KMO value was 0.767, which was greater than 0.7, which passed Bartlett’s sphericity test, indicating that the questionnaire is particularly valid.

### Common method bias test

This paper used Harman’s one-way test to examine and assess common method bias by conducting exploratory factor analysis on all items involved in all variables in the study (travel advertisement image-text interaction pattern, perceived advertising effectiveness, destination affective image, and visit intention) with the SPSS software. The results of the Harman unrotated exploratory factor analysis showed that three factors with eigenvalues greater than 1 were formed, and the variance explained by the first factor when unrotated was found to be 38.763%, which is less than the critical value of 40%, indicating that there is no serious common method bias in this study. The results are shown in Supplementary Appendix Table S3.

### Multi-factor analysis of variance

#### Overview of impacts of different presentation forms of the scenic spot’s name in tourism advertisements

The five-point Likert scale was adopted in questionnaires and a theoretical mean value of 3 is regarded as a reference value for the overall analysis of the variables. As shown in Supplementary Appendix Table S4, the perceived advertising effectiveness has a minimum value of 1.250 and a maximum value of 5. The mean value is 3.274, higher than the reference value, indicating that the overall level of tourists’ perceived advertising effectiveness of travel advertisements for the tourist destination city in this study is high. The minimum value of destination affective image score is 1.75, the maximum value is 5, and the mean value is 3.686, higher than the reference value, indicating that the overall level of tourists’ destination affective image of tourism advertisements for the tourist destination city is high. The minimum value of visit intention is 1.5, the maximum value is 4.75, and the mean value is 3.527, higher than the reference value, indicating that there is a strong visit intention to the tourist destination city.

#### Multi-factor analysis of variance

First, by the Levene’ s test for homogeneity of error variance (Tables 1, 2), when the dependent variable is the perceived advertising effectiveness (*p* = 0.884 > 0.05), the test is passed. When the dependent variable is the destination affective image (*p* = 0.983 > 0.05), passing the test. When the dependent variable is the visit intention (*p* = 0.080 > 0.05), passing the test. In summary, it indicates that a multi-factor ANOVA can be conducted.

**Table 1:** Scales source.

Variables	Items	Sources
Perceived advertising effectiveness	This photo evokes my interest in this place I would like to visit this place more times This photo is conducive to decision making in travel This photo contains useful information	Hwang and Hyun (2016); Li et al. (2016); Jafarzadeh et al. (2019); Lee et al. (2019); Van den Broeck et al. (2019)
Destination affective image	Unpleasant-pleasant Drowsy-exhilarating Depressing-exciting Dreary-relax	Russell (1980); Tosun et al. (2015); Woosnam et al. (2020); Kim et al. (2017)
Visit intention	I may travel to Jiujiang in the future I plan to travel to Jiujiang in the future I will recommend Jiujiang to my friends and relatives in the future	Woodside et al. (1989); Shen et al. (2009); Liang and Lai (2022)

Source: Produced by the author.

**Table 2:** Levene’s test for homogeneity of error variance.

	*F*	df1	df2	*p*	Result
Perceived advertising effectiveness	0.218	3	102	0.884	Passed
Destination affective image	0.056	3	102	0.983	Passed
Visit intention	2.322	3	102	0.080	Passed

##### Perceived advertising effectiveness

The main effect of image presentation form was not significant (*F* = 0.073, *p* > 0.05), the main effect of text presentation form was significant (*F* = 7.094, *p* < 0.01), and the interaction effect of image presentation form and text presentation form was significant (*F* = 4.222, *p* < 0.05), indicating that whether the image part contains the scenic spot’s name or not, it has no significant impact on perceived advertising effectiveness, rejecting H1a. The text part has a significant impact on perceived advertising effectiveness, supporting H1b. In the pairwise comparison analysis by text presentation form (shown in Tables 3, 4), perceived advertising effectiveness scored higher when the text part contained the scenic spot’s name than when the text part did not contain the scenic spot’s name (I-J = 0.372, *p* < 0.01). The results of the pairwise comparison of the interaction of image presentation×text presentation (shown in Table 5) showed that the interaction effect was not significant when the image part did not contain the scenic spot’s name, regardless of whether the text part containing the scenic spot’s name or not. When the image part contains the scenic spot’s name, the perceived advertising effectiveness scores higher and the difference is significant if the text part contains the scenic spot’s name than without containing the scenic spot’s name (I-J = 0.659, *p* < 0.01).

**Table 3:** Multi-factor analysis of variance.

Variable	Source of variation	III sum of square	df	Mean square	*F*	*p*
Perceived advertising effectiveness	Modified model	6.082	3	2.027	3.931*	0.011
	Intercept	1130.237	1	1130.237	2191.346***	0.000
	Image presentation form	0.502	1	0.502	0.973	0.326
	Text presentation form	3.659	1	3.659	7.094**	0.009
	Image presentation form × Text presentation form	2.178	1	2.178	4.222*	0.042
	Error	52.609	102	0.516		
	Total	1194.625	106			
	Modified total variation	58.691	105			
Destination affective image	Modified model	2.429	3	0.81	2.554	0.06
	Intercept	1434.689	1	1434.689	4526.519	0.000
	Image presentation form	0.161	1	0.161	0.507	0.478
	Text presentation form	0.326	1	0.326	1.027	0.313
	Image presentation form × Text presentation form	2.009	1	2.009	6.34*	0.013
	Error	32.329	102	0.317		
	Total	1475.188	106			
	Modified total variation	34.758	105			
Visit intention	Modified model	4.116a	3	1.372	4.161**	0.008
	Intercept	905.373	1	905.373	2745.999***	0
	Image presentation form	0.025	1	0.025	0.076	0.783
	Text presentation form	2.63	1	2.63	7.976**	0.006
	Image presentation form × Text presentation form	1.599	1	1.599	4.850*	0.030
	Error	33.63	102	0.33		
	Total	945.813	106			
	Modified total variation	37.746	105			

***p < 0.001, **p < 0.01, *p < 0.05.

**Table 4:** Pairwise comparison of text presentation form.

(I) Text presentation form	(J) Text presentation form	Mean difference (I,J)	*SE*	*p*	95% confidence interval	
					Lower limit	Upper limit
Text part without the scenic spot’s name	Text part with the scenic spot’s name	−0.372**	0.14	0.009	−0.649	−0.095
Text part with the scenic spot’s name	Text part without the scenic spot’s name	0.372**	0.14	0.009	0.095	0.649

***p < 0.001, **p < 0.01, *p < 0.05.

**Table 5:** Pairwise comparison of image presentation form× text presentation form.

Image presentation form	(I) Text presentation form	(J) Text presentation form	Difference of mean (I,J)	*SD*	*p*	95% confidence interval	
						Lower limit	Upper limit
Image part without the scenic spot’s name	Text part without the scenic spot’s name	Text part with the scenic spot’s name	−0.085	0.194	0.662	−0.469	0.299
	Text part with the scenic spot’s name	Text part without the scenic spot’s name	0.085	0.194	0.662	−0.299	0.469
Image part with the scenic spot’s name	Text part without the scenic spot’s name	Text part with the scenic spot’s name	−0.659**	0.201	0.001	−1.058	−0.26
	Text part with the scenic spot’s name	Text part without the scenic spot’s name	0.659**	0.201	0.001	0.26	1.058

***p < 0.001, **p < 0.01, *p < 0.05.

##### Destination affective image

The main effect of image presentation form was not significant (*F* = 0.507, *p* > 0.05), nor was the main effect of text presentation form (*F* = 1.027, *p* > 0.05), indicating that whether the image part or text part contained the scenic spot’s name had no significant impact on destination affective image (shown in Table 3), rejecting H2a and H2b. The interaction effect between image presentation form and text presentation form was significant (*F* = 6.340, *p* < 0.05). The results of the pairwise comparison of the interaction of image presentation form× text presentation form (Table 6) showed that when the image part did not contain the scenic area name, no matter whether the text part contained the name of scenic spot or not, the interaction effect was not significant. When the image part contains the scenic spot’s name, the destination affective image scores higher and the difference is significant if the text part contains the scenic spot’s name than without containing the scenic spot’s name (I-J = 0.387, *p* < 0.01).

**Table 6:** Pairwise comparison of Image presentation form × text presentation form.

Image presentation form	(I) Text presentation form	(J) Text presentation form	Difference of mean (I,J)	*SD*	*p*	95% confidence interval	
						Lower limit	Upper limit
Image part without the scenic spot’s name	Text part without the scenic spot’s name	Text part with the scenic spot’s name	0.165	0.152	0.281	−0.137	0.466
	Text part with the scenic spot’s name	Text part without the scenic spot’s name	−0.165	0.152	0.281	−0.466	0.137
Image part without the scenic spot’s name	Text part without the scenic spot’s name	Text part with the scenic spot’s name	−0.387*	0.158	0.016	−0.699	−0.074
	Text part with the scenic spot’s name	Text part without the scenic spot’s name	0.387*	0.158	0.016	0.074	0.699

***p < 0.001, **p < 0.01, *p < 0.05.

##### Visit intention

The main effect of image presentation form was not significant (*F* = 0.076, *p* > 0.05), the main effect of text presentation form was significant (*F* = 7.976, *p* < 0.01), and the interaction effect of image presentation form and text presentation form was significant (*F* = 4.850, *p* < 0.05), indicating that whether the image part contains the scenic spot’s name or not, it has no significant impact on the visit intention. The pairwise comparative analysis of the text section (Supplementary Appendix Table S4–S8) showed that the perceived advertising effectiveness scored higher when the text part contains the scenic spot’s name than when the text part did not (I-J = 0.372, *p* < 0.01). The results of the pairwise comparison of the interaction term image presentation form × text presentation form (Supplementary Appendix Table S4–S9) showed that the interaction effect was not significant when the image part did not contain the scenic spot’s name, regardless of whether the text part containing the scenic spot’s name or not. When the image part contains the scenic spot’s name, tourists’ visit intention scores higher and the difference is significant if the text part contains the scenic spot’s name than without containing the scenic spot’s name (I-J = 0.372, *p* < 0.01).

**Figure 1:**
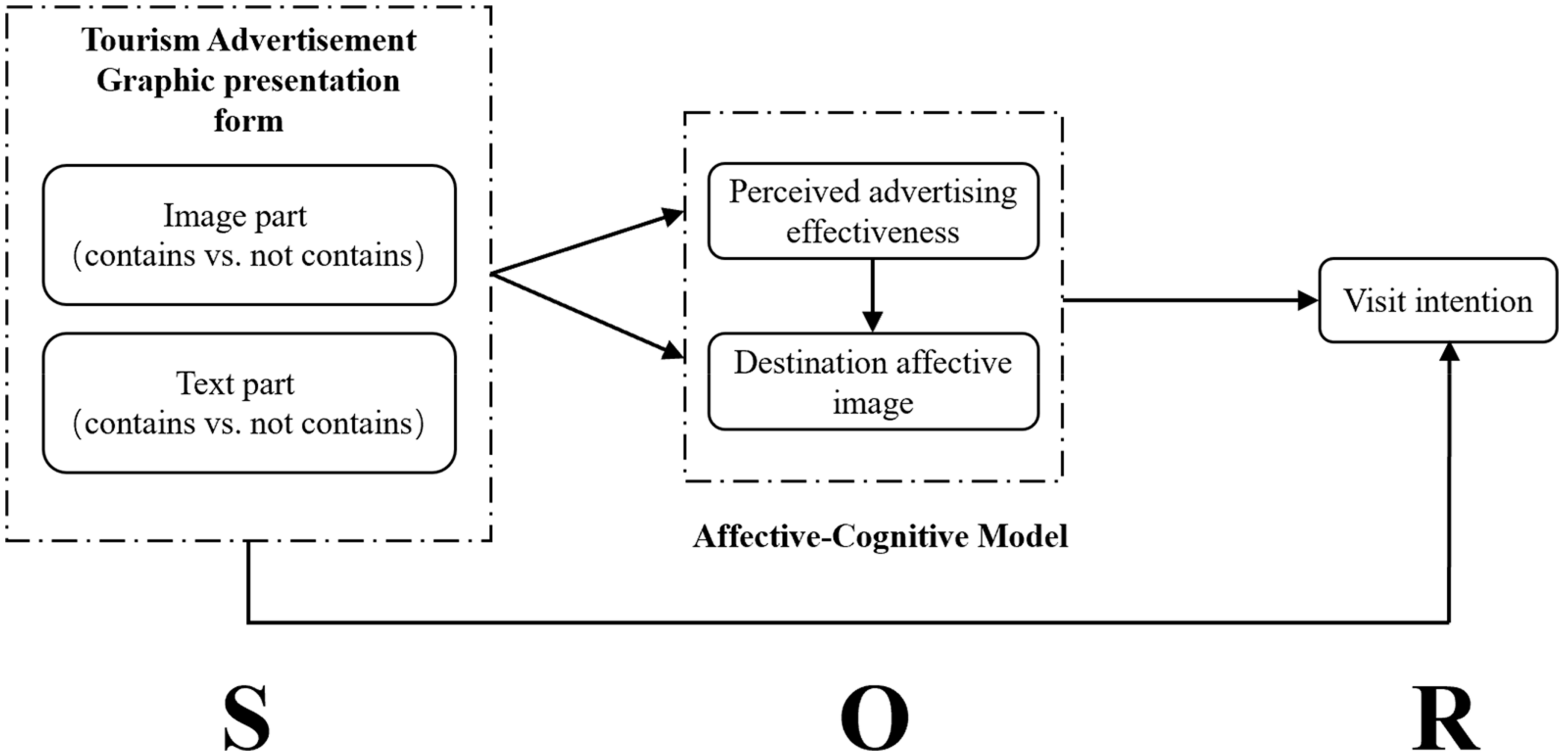
Theoretical framework. Source: Drawn by the author.

**Figure 2:**
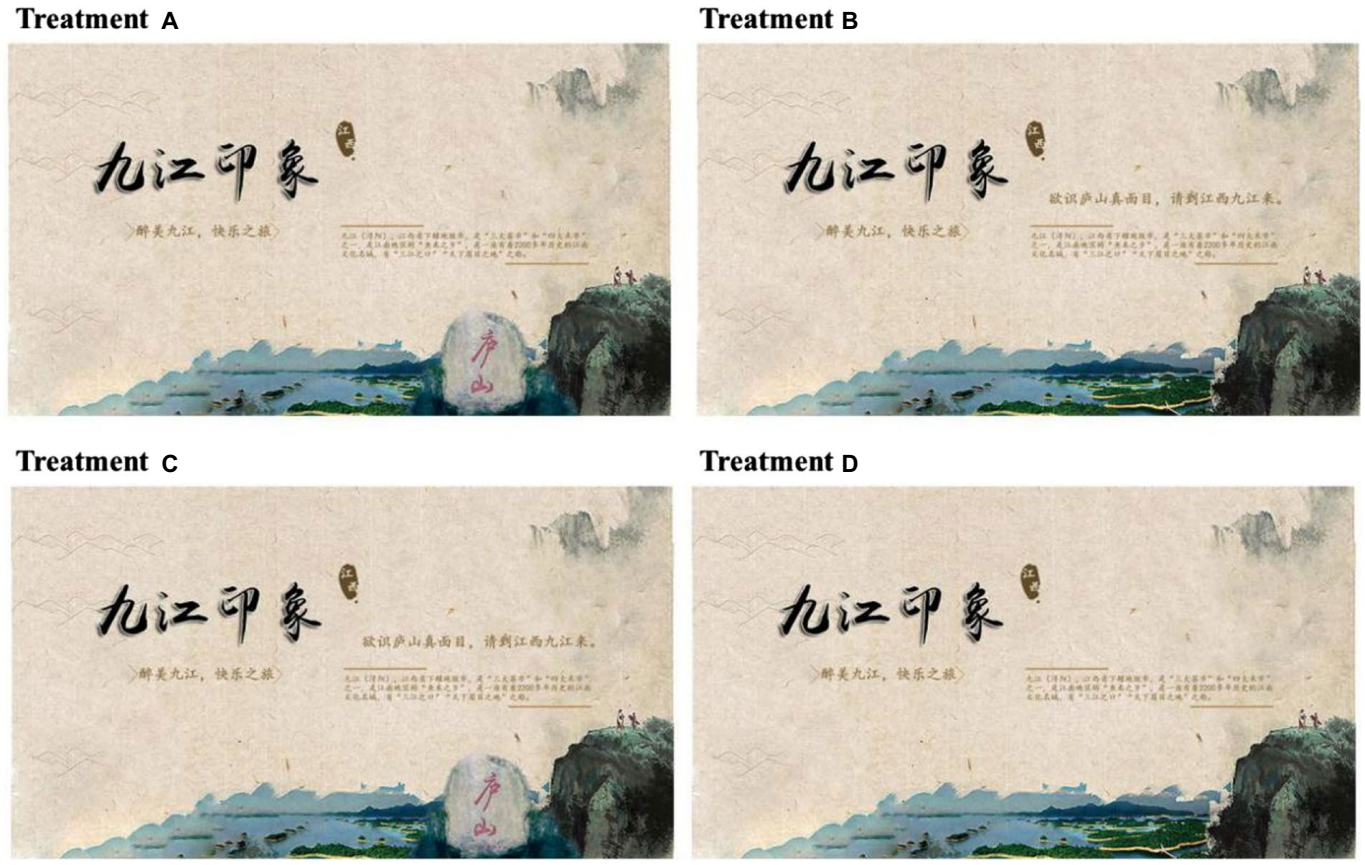
Stimulus materials. Source: made by one of authors using Photoshop software based on the true tourism advertisement. The main body of the advertisements includes image and text parts, and the basic elements of the image part are the ink painting of Mount Lu at the bottom right of the advertisement and the lake at the bottom center. The basic elements of the text part are “Jiujiang Impression,” the slogan that Jiujiang is the most beautiful city in China, and the introduction of Jiujiang City on the right side, which says Jiujiang is a prefecture-level city under Jiangxi Province. Jiujiang is one of the three major tea markets and one of the four major rice markets. It is the hometown of fish and rice in the Jiangnan region as a famous cultural city in Jiangnan with a history of more than 2,200 years.

Moreover, mean scores of perceived advertising effectiveness, destination affective image, and visit intention in different experimental treatment are shown in the Figures 1, 2.

### Mediating effect analysis

A simple mediation model Model 4 in SPSS adopted from Hayes (2012) was used to test the mediating effect between perceived advertising effectiveness, destination affective image in different presentation forms of the scenic spot’s name and the visit intention.

#### Mediating effect of perceived advertising effectiveness

##### Image presentation form

The results of the regression analysis showed that (Supplementary Appendix Table S5), the image presentation form of the scenic spot’s name did not have a significant impact on visit intention (*B* = 0.037, *t* = 0.316, *p* > 0.05). When the mediating variable is perceived advertising effectiveness, the image presentation form of the scenic spot’s name did not have a significant impact on visit intention (*B* = 0.094, *t* = 0.955, *p* > 0.05) and perceived advertising effectiveness (*B* = -0.131, *t* = −0.897, *p* > 0.05). However, perceived advertising effectiveness had a significant impact on visit intention (*B* = 0.440, *t* = 6.627, *p* < 0.01).

According to Supplementary Appendix Table S6, the upper and lower limits of the bootstrap 95% confidence interval for the total, direct, and indirect effects of the image presentation form of the scenic spot’s name on the visit intention to the tourist destination city contain 0, indicating that the impact of the image presentation form of the scenic spot’s name on the visit intention cannot be achieved through the mediating effect of perceived advertising effectiveness, rejecting H7a.

##### Text presentation form

As is shown in Supplementary Appendix Table S5, the text presentation form of the scenic spot’s name had a significant impact on visit intention (*B* = 0.307, *t* = 2.711, *p* < 0.01). When the mediating variable is perceived advertising effectiveness, the text presentation form of the scenic spot’s name had no significant impact on visit intention (*B* = 0.160, *t* = 1.592, *p* > 0.05). The perceived advertising effectiveness had a significant impact on visit intention (*B* = 0.409, *t* = 6.042, *p* < 0.01). The text presentation form of the scenic spot’s name had a significant impact on perceived advertising effectiveness (*B* = 0.359, *t* = 2.531, *p* < 0.05).

According to Supplementary Appendix Table S6, the upper and lower limits of the bootstrap 95% confidence interval of the total and indirect effect of the text presentation form of the scenic spot’s name on the visit intention to the tourist destination city do not contain 0, indicating the total and indirect effects are significant. However, the upper and lower limits of the bootstrap 95% confidence interval of the direct effect of the text presentation of the scenic spot’s name on the visit intention to the tourist destination city contain 0 so the direct effect is not significant, which refers to that the impact of the text presentation form of the scenic spot’s name on the visit intention can be realized through the mediating effect of perceived advertising effectiveness, which plays a complete mediating role, supporting H7b.

#### Mediating effect of destination affective image

##### Image presentation form

According to Supplementary Appendix Table S5, there was no significant impact of the image presentation form of the scenic spot’s name on the visit intention (*B* = 0.037, *t* = 0.316, *p* > 0.05). When the mediating variable is destination affective image, the image presentation form of the scenic spot’s name had no significant impact on visit intention (*B* = 0.069, *t* = 0.637, *p* > 0.05) and destination affective image (*B* = -0.076, *t* = −0.675, *p* > 0.05). Destination affective image had a very significant impact on tourists’ visit intention (*B* = 0.420, *t* = 4.454, *p* < 0.01).

The upper and lower limits of the bootstrap 95% confidence interval of the total, direct, and indirect effects of the image presentation form of the scenic spot’s name on the visit intention to the tourism destination city contain 0 (shown in Supplementary Appendix Table S6). Thus, the total, direct, and indirect effects are not significant, indicating that the impact of the image form of the scenic spot’s name on the visit intention cannot be realized through the mediating effect of the destination affective image, rejecting H8a.

##### Text presentation form

The results of the regression analysis showed that (Supplementary Appendix Table S7), the total effect of the text presentation form of the scenic spot’s name on the visit intention was significant (*B* = 0.307, *t =* 2.711, *p* < 0.01). When the mediating variable is destination affective image, the text presentation form of the scenic spot’s name had a significant impact on visit intention (*B* = 0.267, *t* = 2.547, *p* < 0.05), but had no significant predictive effect on destination affective image (*B* = 0.099, *t* = 0.885, *p* > 0.05). The destination affective image has a significant impact on visit intention (*B* = 0.395, *t* = 4.313, *p* < 0.01).

According to Supplementary Appendix Table S6, the upper and lower limits of bootstrap 95% confidence interval for total and direct effects of the text presentation of the scenic spot’s name on the visit intention to the tourist destination city do not contain 0 so the total and direct effects are significant. Hence, the upper and lower limits of bootstrap 95% confidence interval for the indirect effect of the text presentation form of the scenic spot’s name on the visit intention to the tourist destination city contain 0 so the indirect effect is not significant, indicating that the impact of the text presentation form of the scenic spot’s name on the visit intention cannot be realized through the mediating role of destination affective image, rejecting H8b.

#### Chain mediating effect test

Based on the correlation analysis, it is known that there is a correlation between the perceived advertising effectiveness and the destination affective image so the chain mediation model was used to further test the mechanism of the impact of presentation forms on the visit intention. The chain mediation model Model 6 in SPSS adopted from Hayes (2012) was used to test the chain mediating effect between perceived advertising effectiveness, destination affective image, and visit intention in the form of image presentation form of the scenic spot’s name.

##### Image presentation form

The results of the regression analysis showed that (Supplementary Appendix Table S8), the direct effect of the image presentation form of the scenic spot’s name on the visit intention was not significant (*B* = 0.103, *t* = 1.072, *p* > 0.05) and the total effect was not significant (*B* = 0.037, *t* = 0.316, *p* > 0.05), rejecting H6a.

When the mediating variables are perceived advertising effectiveness and destination affective image, the direct effect of the image presentation form of the scenic spot’s name on the visit intention was not significant (*B* = 0.103, *t* = 1.072, *p* > 0.05). The predictive effect of perceived advertising effectiveness and destination affective image on visit intention were positively significant (*B* = 0.371, *t* = 5.300, *p* < 0.01; *B* = 0.371, *t* = 2.602, *p* < 0.05), supporting H4a and H5a. The predictive effect of the image presentation form of the scenic spot’s name on the perceived advertising effectiveness (*B* = 0.371, *t* = 5.300, *p* < 0.01) and destination affective image (*B* = 0.236, *t* = 2.602, *p* < 0.05) was positively significant. The perceived advertising effectiveness had a significantly positive predictive impact on the destination affective image (*B* = 0.294, *t* = 4.195, *p* < 0.05), supporting H3a.

According to Supplementary Appendix Table S9), the total indirect effect of the image presentation form of the scenic spot’s name on the visit intention was not significant. Specifically, the mediating effect consists of the indirect effects generated by three paths, and the upper and lower limits of the bootstrap 95% confidence interval of all three paths contain 0 so the indirect effects of all three paths are not significant, indicating that the impact of the image presentation form of the scenic spot’s name on the visit intention cannot be achieved through the chain mediating effect of perceived advertising effectiveness and destination affective image, rejecting H9a.

##### Text presentation form

According to Supplementary Appendix Table S10, the total effect of the text presentation form of the scenic spot’s name on the visit intention was positively significant (*B* = 0.037, *t* = 2.711, *p* < 0.01), supporting H6b. The direct effect of the text presentation form of the scenic spot’s name on the visit intention was not significant (*B* = 0.162, *t* = 1.654, *p* > 0.05). The text presentation form of the scenic spot’s name had a significant impact on the perceived advertising effectiveness (*B* = 0.359, *t* = 2.531, *p* < 0.05).

When the mediating variables are perceived advertising effectiveness and destination affective image, the predictive effect of the text presentation form of the scenic spot’s name on the visit intention was not significant (*B* = 0.162, *t* = 1.654, *p* > 0.05). The text presentation form of the scenic spot’s name had a significant impact on perceived advertising effectiveness and destination affective image (*B* = 0.339, *t* = 4.770, *p* < 0.01; *B* = 0.234, *t* = 2.597, *p* < 0.05), supporting H4b and H5b. The predictive impact of the image presentation form of the scenic spot’s name on the destination affective image was not significant (*B* = -0.008, *t* = −0.072, *p* > 0.05) but significant for perceived advertising effectiveness (*B* = 0.359, *t =* −2.531, *p* < 0.05). Perceived advertising effectiveness had a significant impact on destination affective image (*B* = 0.298, *t* = 4.132, *p* < 0.01), supporting H3b.

According to Supplementary Appendix Table S11, the upper and lower limits of the bootstrap 95% confidence interval of the total indirect effect of the text presentation form of the scenic spot’s name on the visit intention do not contain 0, indicating that the total indirect effect is significant. Moreover, the mediating effect consists of the indirect effect generated by the three paths, whose upper and lower limits of the bootstrap 95% confidence intervals do not contain 0, indicating that the mediating effect is significant. In brief, the impact of the text presentation form of the scenic spot’s name on the visit intention can be realized through the mediating effect of perceived advertising effectiveness and destination affective image, supporting H9b.

## Eye movement data analysis

### Correlation analysis between eye-movement data and perceived advertising effectiveness, destination affective image, and visit intention

By using EyeSo Studio software to analyze the eye-movement data, we can obtain the total number of fixation counts, total fixation duration, average fixation duration, and fixation coordinates of the participants from four groups. Each advertisement can be divided into text part and image part by dividing the area of interest (AOI). After dividing the AOI, we can get data including: the total fixation counts, the fixation duration, the average fixation duration in the text and image part, respectively.

Pearson’s correlation analysis was conducted with perceived advertising effectiveness, destination affective image, visit intention, and nine eye-movement indicators including: total fixation counts, total fixation duration, average fixation duration, total fixation counts in the text part, the fixation duration in the text part, the average fixation duration in the text part, total fixation counts in the image part, the fixation duration in the image part, and the average fixation duration in the image part (shown in Table 7). The results of the correlation analysis show that there is a negative weak correlation between the total fixation counts and perceived advertising effectiveness (*p* = 0.006 < 0.05) and visit intention (*p* = 0.039 < 0.05). The correlation coefficients are-0.227 and-0.209 respectively, and the absolute values of coefficients are between 0.2 and 0.4, indicating that the more fixation counts in the image part of the tourism advertisement, the weaker the tourists’ perceived advertising effectiveness and visit intention.

**Table 7:** Correlation analysis result.

		Total fixation count	Total fixation duration	Average fixation duration	Total fixation count in text part	Total fixation duration in text part	Average fixation duration in text part	Total fixation count in image part	Total fixation duration in image part	Average fixation duration in image part
Perceived advertising effectiveness	(Pearson) Correlation	−0.097	−0.097	0.097	0.129	0.19	0.144	−0.277**	−0.199	0.115
	Significance (two-tailed)	0.345	0.345	0.344	0.207	0.062	0.161	0.006	0.051	0.264
Destination affective image	(Pearson) Correlation	−0.019	0.038	0.006	0.093	0.083	0.011	−0.146	−0.079	0.218*
	Significance (two-tailed)	0.854	0.712	0.953	0.366	0.418	0.912	0.153	0.442	0.032
Visit intention	(Pearson) Correlation	−0.136	−0.054	0.109	0.049	0.179	0.204*	−0.209*	−0.184	0.222*
	Significance (two-tailed)	0.183	0.601	0.288	0.633	0.079	0.045	0.039	0.072	0.028

***p < 0.001, **p < 0.01, *p < 0.05.

The correlations between the average fixation duration of the text part and the destination affective image (*p* = 0.032) and visit intention (*p* = 0.045) were less than 0.05. The correlation coefficients were 0.218 and 0.222, respectively, so the absolute values of the coefficients were between 0.2 and 0.4, indicating that there is a positive weak correlation between the total fixation duration and the perceived advertising effectiveness as well as visit intention. Thus, the longer the average fixation duration of the text part of the tourism advertisement, the stronger the tourists’ destination affective image and visit intention.

### Eye-movement heat map analysis

**Figure 3:**
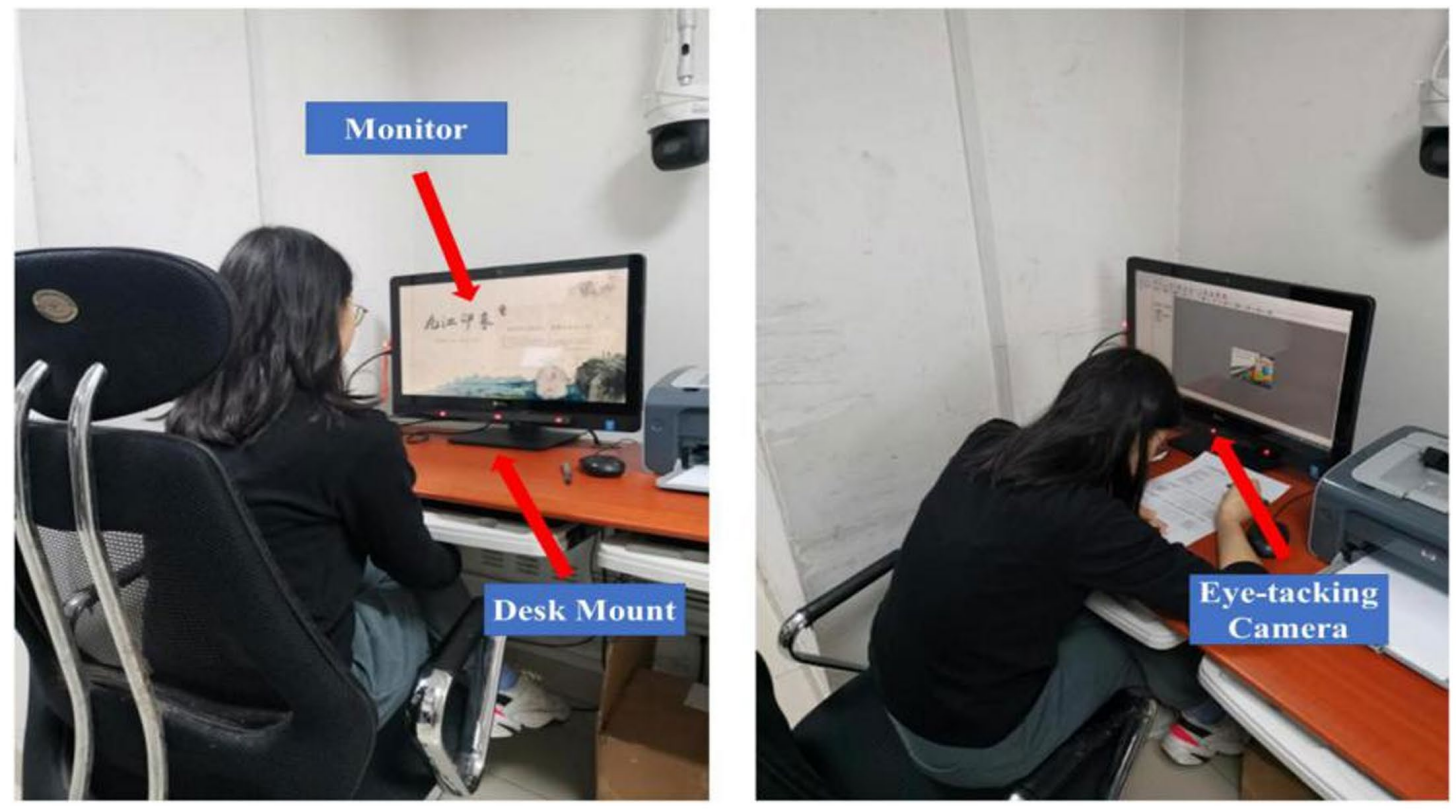
Experiment procedure. Source: photographed by research assistants.

**Figure 4:**
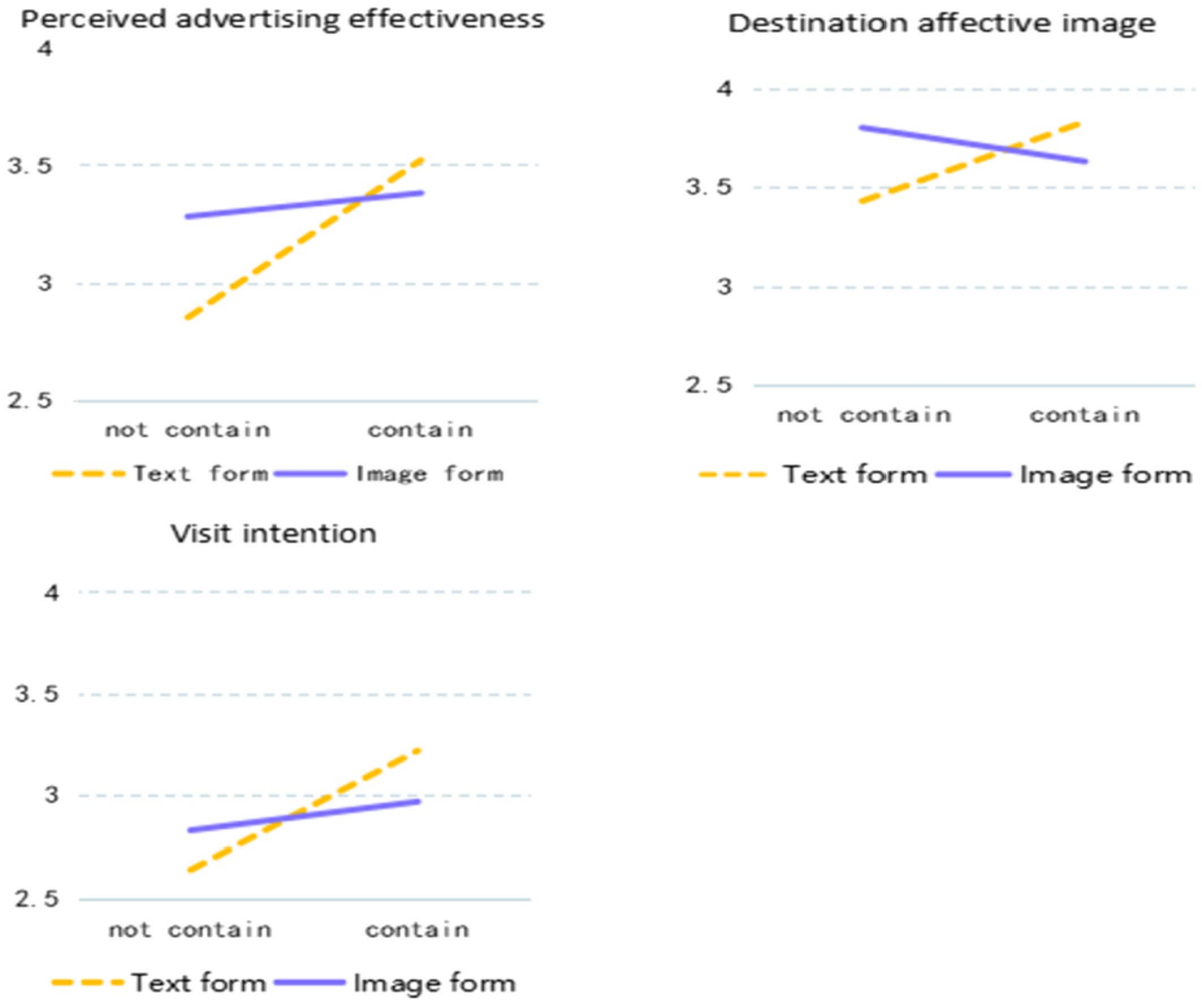
Mean value for perceived advertising effectiveness, destination affective image, and visit intention. Source: Made by the Author.

**Figure 5:**
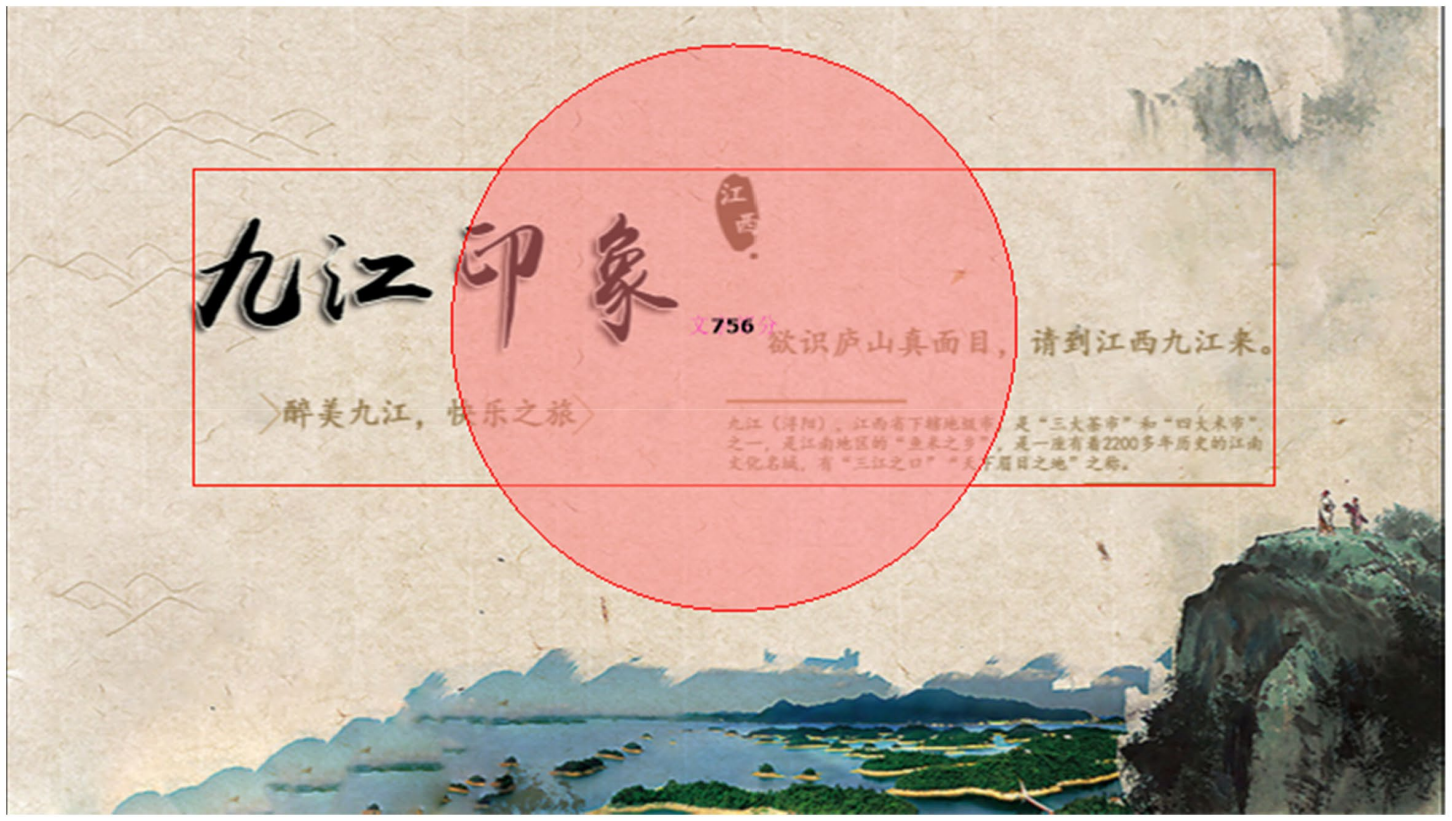
Division of area of interest. Source: EyeSo Studio software.

**Figure 6:**
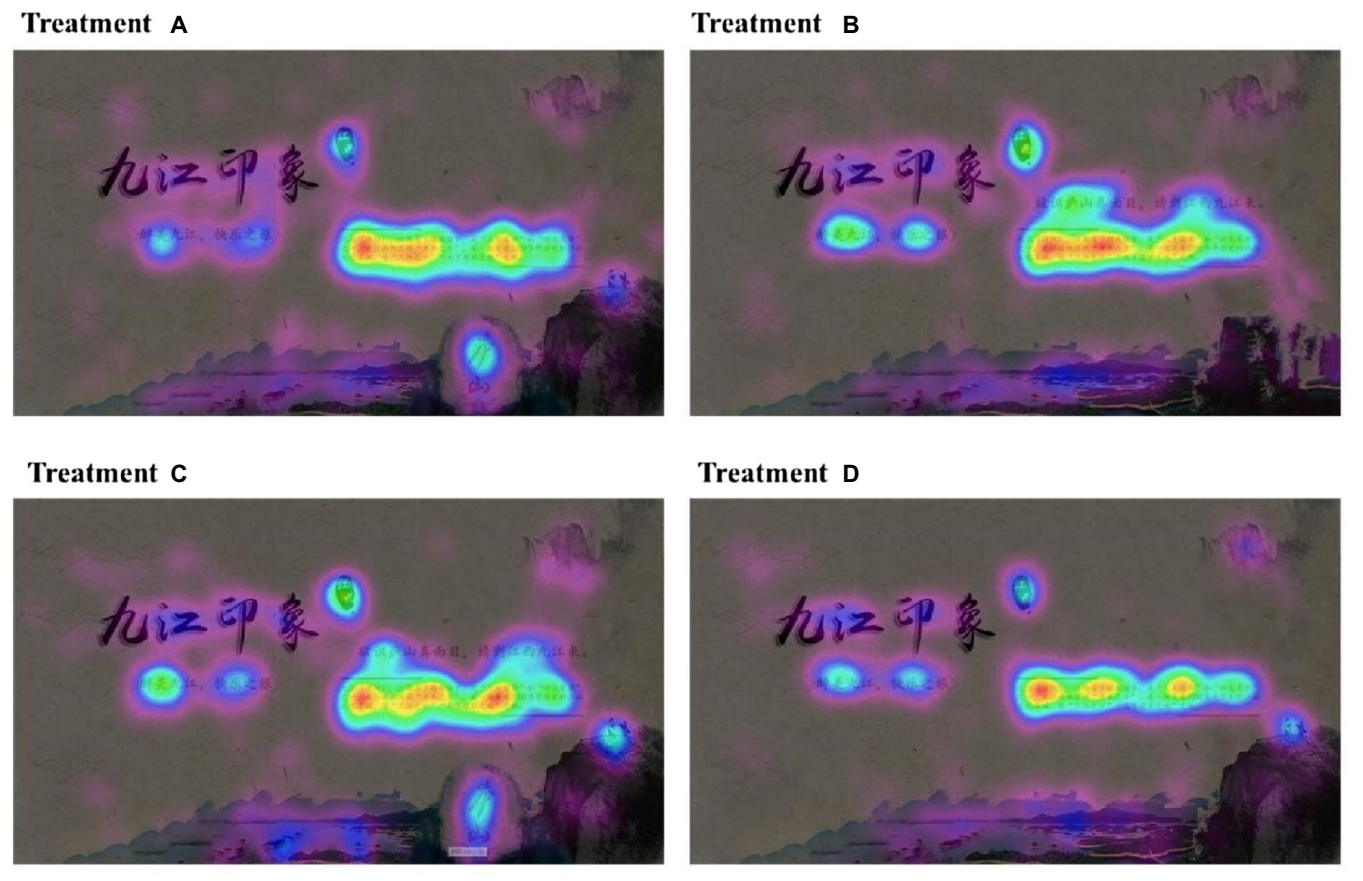
Heat map for eye movement. Source: EyeSo Studio software.

The eye-movement heat map Figures 3–6) can clearly visualize the distribution of the participant’s fixation through the color distribution. The heat map uses a rainbow map in this study. The extent of participant’s fixation gradually decreases in accordance with color changing from red, orange, yellow, green, cyan, blue, to purple. The closer the color is approaching red, the more the participants watch the area and the longer the fixation duration. The closer the color is approaching purple, the less the participants watch the area and the shorter the fixation duration. Watching the advertisements, participants focus more on the area where the text was more concentrated, like the city introduction of Jiujiang City, “Jiangxi province,” “the home of fish and rice in Jiangnan area,” “one of three major tea market” and so on, followed by the slogan of “enchanted by Jiujiang, enjoying happy trips.”

In the heat map of group B and group C with the slogan “To know the true face of Mount Lu, please come to Jiujiang, Jiangxi province,” participants focused more on the words “Mount Lu” and “Jiujiang, Jiangxi,” indicating that the slogan can not only attract the participants’ attention but also remind them that Mount Lushan is located in Jiujiang, Jiangxi province. Thus, participants can associate the scenic spot with the city. In groups A and C, the word “Lushan” engraved on a stone tablet, the participants also concentrated more on the word “Lushan.”

To draw a conclusion, participants concentrate more on the text part when viewing the advertisements and the information contained in the text part. In the textual content, participants pay more attention to the names of regions, scenic spots, and descriptions of the destination city, such as “Jiangxi,” “Jiujiang,” “the home of fish and rice in the Jiangnan area,” “the confluence of three rivers,” etc.

## Conclusion and discussion

### Conclusion

Tourism advertisement marketing has long been one of the focuses of the academic circle. This study investigates the impact of different presentation forms of tourism advertisements on tourists’ visual attention, perceived advertising effectiveness, destination affective image, and visit intention.

First of all, from the experimental results, when the tourism advertisement is presented in the image form, the main effect is not significant no matter whether the dependent variable is perceived advertising effectiveness, destination affective image, or visit intention, but the interaction effect between image presentation form and text presentation form is significant. The scores of perceived advertising effectiveness, destination affective image, and visit intention are higher when the text part contains the scenic spot’s name than when the text part does not.

When the tourism advertisement is presented in the text form, the main effect is significant when the dependent variable is perceived advertising effectiveness and visit intention, while the main effect is not significant when the dependent variable is destination affective image.

Secondly, in the mediation test, we discovered that when the scenic spot’s name in tourism advertisements is presented in text form, its impact on the visit intention can be realized through the mediating role of perceived advertising effectiveness. Furthermore, when the scenic spot’s name is presented in text form in tourism advertisements, its impact on the visit intention can be realized through the multiple mediating effects of perceived advertising effectiveness and destination affective image.

Finally, three findings can be obtained from the eye-tracking analysis: 1. When participants watched tourism advertisements, their fixation counts were more concentrated in the text part. That’s to say, participants pay more attention to the information contained in the text part. 2. This study found that there is a negative and weak correlation between the total fixation counts in the image part of tourism advertisements and the perceived advertising effectiveness and visit intention, indicating that the more fixation counts in the image part of the tourism advertisement, the weaker the perceived advertising effectiveness and visit intention. 3. There was a positive and weak correlation between the total fixation counts in the image part and the destination affective image and visit intention, indicating that the longer tourists gaze at the text part of the advertisement, the stronger the impact on the destination affective image and visit intention of the destination.

### Discussion

#### Theoretical implications

Our theoretical contributions are mainly in three aspects. First of all, based on a literature review in the field of tourism and hospitality research, by investigating the impact of different presentation forms of real tourist destination marketing advertisements on tourists’ perceived advertising effectiveness, destination affective image, and visit intention, this paper is the pioneer of experimental research on this aspect. It is in response to the study of Viglia and Dolnicar (2020), who advocated that academic researchers in the field of tourism and hospitality should actively adopt experimental methods to explore the causal relationships within academic issues. To the best of our knowledge, picture and text marketing strategies are widely used in tourist destination marketing advertisements. On the one hand, previous scholars have studied the impact of different text advertisements such as textual expression, different sources, text language, text and advertisement matching, etc (Byun and Jang, 2015; Kim and Jang, 2019; Deng, 2020; Delmelle and Nilsson, 2021) to confirm the role of text in effective information dissemination in advertisements. On the other hand, the marketing impact of picture application in tourism advertisements has also been studied and it can be summarized as “a picture is worth a thousand words” (Leung et al., 2017; Del Gesso, 2021; Luo et al., 2021). Many scholars believe that pictures can quickly catch the eye of consumers in tourism advertisements and increase their attention to tourism advertisements. But until now, these two types of different presentation forms have been ignored by academic researchers in the field of tourism and hospitality. Understandings of the current tourism advertisements can be deepened by investigating the different presentation forms of images and texts of tourist destination marketing advertisements.

Second, given that constructivism cognitive psychology theory, this study integrated SOR theoretical framework and affective-cognitive decision-making model to investigate the multiple mediating effects of perceived advertising effectiveness and destination affective image. Previous studies only explored the perceived advertising effectiveness (Cham et al., 2022) or the destination affective image (Tosun et al., 2015; Papadimitriou et al., 2015) as a mediator. Based on the dual processing paths of information in cognitive psychology and the affective-cognitive decision-making model, this study put the perceived advertising effectiveness and the destination affective image in the same research framework, and further elucidated the potential mechanism of different presentation forms in destination marketing. It enriched the theory of constructivism in cognitive psychology and complemented the empirical research in this field.

Finally, there are many scenic spots with a high degree of public familiarity, but the public is not familiar with the city (tourist destination city), forming a “cover effect.” However, previous studies almost only focused on the visit intention of scenic spots and ignored the visit intention of tourist destination cities. In this study, eye-tracking technology was used to perform a visual analysis of scenic spots with high familiarity and tourist destination cities with low familiarity, which bridges the gap in the literature in this field. The results of this study provide suggestions for developing effective visual marketing materials to attract the visual attention of tourists and elicit positive attitudes towards tourism advertisements and tourist destination cities. Moreover, the application of eye-tracking data in this paper can be regarded as a trigonometric method to provide more reliable results and conclusions. Meanwhile, it also responds to the proposal of Vadalkar et al. (2021) that psychological and physiological analysis tools such as eye trackers should be adopted in advertisement marketing research to obtain more credible results. Eye-tracking can directly capture the eye movement of subjects and record their physiological reactions. The technology also reduces the potential confounding effects caused by social expectation bias and impression management motive when participants fill in questionnaires, thus providing a relatively objective and reasonable method.

#### Practical implications

First of all, destination managers can set about promoting tourists’ destination affective image occurred after they watch tourism advertisements when it comes to using tourism advertisements to carry out marketing promotion of tourist destinations, and further optimize the tourists’ destination affective image. This study has found that different presentation forms have a different impact on destination affective image and visit intention. When the image part and text part of the tourism advertisements both contain the scenic spot’s name of the destination city, tourists have the strongest willingness to visit the city. Therefore, destination city managers should pay more attention to applying core scenic elements in destination cities to designing tourism advertisements for destination cities.

Second, the great importance should be attached to core scenic spots for shaping the whole image of the tourist destination city shape. The image and text presentation can highlight the relationship between scenic spots and the corresponding destination cities so as to generate an interactive effect, which can enable tourists to have an emotional link between scenic spots and tourist destination cities. The positive emotion to scenic spots that tourists have can spread to the destination city where the scenic spot is located, resulting in the emotional effect. Destination cities should take full advantage of the popularity of the renowned scenic spots to achieve radiation effects as well as the effect of “love me, love my dog” to help the destination city form a good image. Tourism advertisement design can be reinforced by the image and text presentation jointly to carry out tourist destination marketing, which is conducive to altering the phenomenon that tourists are particularly familiar with the scenic spot while tourists do not know the city where the scenic spot is located. It is of great significance to engender the “bundling marketing” effect between the scenic spot and the destination city so as to improve tourists’ willingness to visit the destination city.

Finally, it can be speculated from the eye-movement analysis that tourists may focus greatly on the text part and attach more importance to the information contained in the text part when watching tourism advertisements. Compared with the image of the scenic spot seen in tourism advertisements, tourists are more inclined to allocate more attention to the text, such as the name of the region, the name of the scenic spot, evaluation phrases of the destination city, propaganda slogan, etc., and combine the information of the text and image to make decisions conjointly. Therefore, based on these findings, the resonance of the text part and the image part is terrifically crucial. The mutual highlight and illustration between the text part and the image part can enhance tourists’ interest in the destination city and facilitate tourists’ visit intention. Because this study is based on real destination marketing advertising. Therefore, the conclusion will be instructive for destination managers to formulate marketing strategies, especially in the era of COVID-19 normalization.

### Limitations and future prospects

Although this study is complementary to previous studies in terms of both theory and practice, there are limitations. First, the present study conducted an eye-tracking experiment in a laboratory setting. The laboratory experiment can avoid irrelevant disturbance, but it cannot simulate the real environment and therefore lacks external validity. In the future, field experiments will be conducted in a real environment and wearable eye-tracking devices can be used to explore visitors’ viewing behavior. Second, though the experiment was conducted rigorously, the participants we recruited were mainly undergraduate and MBA students from China due to the individual contact limitations brought about by the raging covid-19. They can be considered good representatives of Generation Z, but not be fully representative of the entire society. Future studies could further use tourists from other countries as subjects. Third, this study uses real online marketing materials for tourism which only contain text and image elements. With the swift advancement of 5G and ICT technologies, it is expected that animation, VR videos, and AR videos in tourism marketing can be utilized to provide dynamic visual experiences for tourists. Future visual research in the tourism and hospitality field could make full use of eye-tracking technology to study specific research topics.

## Data availability statement

The original contributions presented in the study are included in the article/Supplementary material, further inquiries can be directed to the corresponding author.

## Ethics statement

Ethical review and approval was not required for the study on human participants in accordance with the local legislation and institutional requirements. The patients/participants provided their written informed consent to participate in this study.

## Author contributions

XH provided the conceptualization of this research. WY completed the original draft preparation. WY, QC, and JX completed the data analysis. XH provided the funding and supervision. MX and JS completed the visualization. WY and JX completed experiment procedure. WY and QC completed major modifications. WY and QC completed the method part. JS completed acquisition of data. All authors contributed to the article and approved the submitted version.

## Funding

This study was supported by National Natural Science Foundation of China (No. 41871138) and Shandong University 20QNQT019 Key R & D Plan of Shandong Province (Major Scientific and Technological Innovation Project) 2020cxgc010904).

## Conflict of interest

The authors declare that the research was conducted in the absence of any commercial or financial relationships that could be construed as a potential conflict of interest.

## Publisher’s note

All claims expressed in this article are solely those of the authors and do not necessarily represent those of their affiliated organizations, or those of the publisher, the editors and the reviewers. Any product that may be evaluated in this article, or claim that may be made by its manufacturer, is not guaranteed or endorsed by the publisher.
