# Supplementary material for: Image and text presentation forms in destination marketing: An eye-tracking analysis and a laboratory experiment
Source: Front Psychol. 2022 Dec 2;13:1024991. doi: 10.3389/fpsyg.2022.1024991 (PMC9758203; doi:10.3389/fpsyg.2022.1024991)
Supplement: Supplementary file 1 [file Data_Sheet_1.docx]

Supplementary materials for Image and Text Presentation Form in Destination Marketing: an Eye-tracking Analysis and a Laboratory Experiment

Appendix

3. Research Design and Data Collection

Table3-1 Questionnaire design dimension and items

| Dimensions | Items |
| --- | --- |
| Experience Familiarity | The number of visits |
|  |  |
| Information Familiarity | The number of ways to get information about this landscape |
|  | The number of ways to get information about this city |
|  | Whether to know the tourist destination is in the city |
| Proximity Familiarity | With or without friends or relatives |
| Demographics | Age |
|  | Gender |
|  | Education Background |
|  | Native Place |
|  | Home Address |

Table 3-2 Destination city and Scenic spot alternative

| City | Scenic spot |
| --- | --- |
| Beijing | Summer Palace |
| Hangzhou | West Lake Scenic Spot |
| Taian | Mountain Tai |
| Jiujiang | Mountain Lu |
| Yancheng | Habitat of Migratory Birds |
| Shiyan | Mountain Wudang |
| Chengdu | Mount Qingcheng |
| Datong | Yungang Grottoes |
| Anyang | Yinxu Scenic Spot |
| Xiamen | Gulangyu Island |

1. Data Analysis

Table 4-1 Principal component analysis results without rotation

| Component | eigenvalue | | | Extraction Sums of Squared Loadings | | |
| --- | --- | --- | --- | --- | --- | --- |
|  | Total | Variance% | Accumulation % | Total | Variance % | Accumulation % |
| 1 | 4.264 | 38.763 | 38.763 | 4.264 | 38.763 | 38.763 |
| 2 | 1.487 | 13.518 | 52.281 | 1.487 | 13.518 | 52.281 |
| 3 | 1.328 | 12.074 | 64.355 | 1.328 | 12.074 | 64.355 |
| 4 | 0.949 | 8.627 | 72.982 |  |  |  |
| 5 | 0.818 | 7.438 | 80.420 |  |  |  |
| 6 | 0.537 | 4.883 | 85.303 |  |  |  |
| 7 | 0.447 | 4.059 | 89.363 |  |  |  |
| 8 | 0.387 | 3.517 | 92.879 |  |  |  |
| 9 | 0.309 | 2.812 | 95.692 |  |  |  |
| 10 | 0.252 | 2.294 | 97.985 |  |  |  |
| 11 | 0.222 | 2.015 | 100.000 |  |  |  |

Extraction method: principal component analysis

Table 4-2 Description statistical analysis of perceived advertising effectiveness

|  | N | Minimum(M) | Maximum(X) | Average(E) | SE |
| --- | --- | --- | --- | --- | --- |
| Perceived advertising effectiveness | 106 | 1.250 | 5 | 3.274 | 0.748 |
| Destination affective image | 106 | 1.75 | 5 | 3.686 | 0.575 |
| Visit intention | 106 | 1.5 | 4.75 | 3.527 | 0.600 |

4.5 Mediating effect analysis

Table 4-12 Mediating model of perceived advertising effectiveness

| Presentation form | Regression equation | | Fit indicator | | | Coefficient significance | | |
| --- | --- | --- | --- | --- | --- | --- | --- | --- |
| Image presentation form | Outcome variable | Predictive variable | R | R^2^ | F | B | t | p |
|  | Visit intention |  | 0.031 | 0.001 | 0.100 |  |  |  |
|  |  | Image presentation form |  |  |  | 0.037 | 0.316 | 0.753 |
|  | Perceived advertising effectiveness |  | 0.088 | 0.008 | 0.805 |  |  |  |
|  |  | Image presentation form |  |  |  | -0.131 | -0.897 | 0.372 |
|  | Visit intention |  | 0.547 | 0.300 | 22.031 |  |  |  |
|  |  | Image presentation form |  |  |  | 0.094 | 0.955 | 0.342 |
|  |  | Perceived advertising effectiveness |  |  |  | 0.440 | 6.627*** | 0.000 |
| Text presentation form | Outcome variable | Predictive variable | R | R^2^ | F | B | t | p |
|  | Visit intention |  | 0.257 | 0.066 | 7.349 |  |  |  |
|  |  | Text presentation form |  |  |  | 0.307 | 2.711** | 0.008 |
|  | Perceived advertising effectiveness |  | 0.241 | 0.058 | 6.407 |  |  |  |
|  |  | Text presentation form |  |  |  | 0.359 | 2.531* | 0.013 |
|  | Visit intention |  | 0.557 | 0.310 | 23.179 |  |  |  |
|  |  | Text presentation form |  |  |  | 0.160 | 1.592 | 0.115 |
|  |  | Perceived advertising effectiveness |  |  |  | 0.409 | 6.042*** | 0.000 |

Note.***: p＜.001, **: p＜0.01, *: p＜0.05

4-13 Total effect, direct effect, and indirect effect decomposition table

|  |  |  | Effect value | Boot SE | Boot LLCI | Boot ULCI |
| --- | --- | --- | --- | --- | --- | --- |
| Perceived advertising effectiveness | Image presentation form | Total effect | 0.037 | 0.117 | -0.195 | 0.269 |
|  |  | Direct effect | 0.094 | 0.099 | -0.102 | 0.291 |
|  |  | Mediating effect of perceived advertising effectiveness | -0.057 | 0.066 | -0.200 | 0.059 |
|  | Text presentation form | Total effect | 0.307 | 0.113 | 0.082 | 0.531 |
|  |  | Direct effect | 0.160 | 0.101 | -0.039 | 0.360 |
|  |  | Mediating effect of perceived advertising effectiveness | 0.146 | 0.061 | 0.039 | 0.282 |
| Destination affective image | Image presentation form | Total effect | 0.037 | 0.117 | -0.195 | 0.269 |
|  |  | Direct effect | 0.069 | 0.108 | -0.145 | 0.283 |
|  |  | Mediating effect of destination affective image | -0.032 | 0.049 | -0.148 | 0.055 |
|  | Text presentation form | Total effect | 0.307 | 0.113 | 0.082 | 0.531 |
|  |  | Direct effect | 0.267 | 0.105 | 0.059 | 0.476 |
|  |  | Mediating effect of destination affective image | 0.039 | 0.045 | -0.045 | 0.135 |

Note.***: p＜.001, **: p＜0.01, *: p＜0.05

Table 4-14 Mediating model of destination affective image

| Presentation form | Regression equation | | Fit indicator | | | Coefficient significance | | |
| --- | --- | --- | --- | --- | --- | --- | --- | --- |
| Image presentation form | Outcome variable | Predictive variable | R | R^2^ | F | B | t | p |
|  | Visit intention |  | 0.031 | 0.001 | 0.100 |  |  |  |
|  |  | Image presentation form |  |  |  | 0.037 | 0.316 | 0.753 |
|  | Destination affective image |  | 0.066 | 0.004 | 0.455 |  |  |  |
|  |  | Image presentation form |  |  |  | -0.076 | -0.675 | 0.501 |
|  | Visit intention |  | 0.403 | 0.162 | 9.979 |  |  |  |
|  |  | Image presentation form |  |  |  | 0.069 | 0.637 | 0.526 |
|  |  | Destination affective image |  |  |  | 0.420 | 4.454*** | 0.000 |
| Text presentation form | Outcome variable | Predictive variable | R | R^2^ | F | B | t | p |
|  | Visit intention |  | 0.257 | 0.066 | 7.349 |  |  |  |
|  |  | Text presentation form |  |  |  | 0.307 | 2.711* | 0.008 |
|  | Destination affective image |  | 0.087 | 0.008 | 0.784 |  |  |  |
|  |  | Text presentation form |  |  |  | 0.099 | 0.885 | 0.378 |
|  | Visit intention |  | 0.457 | 0.209 | 13.596 |  |  |  |
|  |  | Text presentation form |  |  |  | 0.267 | 2.547* | 0.012 |

Table 4-15 Chain mediation model of perceived advertising effectiveness and destination affective image

| Regression equation | | Fit indicator | | | Coefficient significance | | |
| --- | --- | --- | --- | --- | --- | --- | --- |
| Outcome variable | Predictive variable | R | R^2^ | F | B | t | p |
| Visit intention |  | 0.031 | 0.001 | 0.100 |  |  |  |
|  | Image presentation form |  |  |  | 0.037 | 0.316 | 0.753 |
| Perceived advertising effectiveness |  | 0.088 | 0.008 | 0.805 |  |  |  |
|  | Image presentation form |  |  |  | -0.131 | -0.897 | 0.372 |
| Destination affective image |  | 0.387 | 0.150 | 9.061 |  |  |  |
|  | Image presentation form |  |  |  | -0.037 | -0.356 | 0.722 |
|  | Perceived advertising effectiveness |  |  |  | 0.294 | 4.195*** | 0.000 |
| Visit intention |  | 0.586 | 0.343 | 17.766 |  |  |  |
|  | Image presentation form |  |  |  | 0.103 | 1.072 | 0.286 |
|  | Perceived advertising effectiveness |  |  |  | 0.371 | 5.300*** | 0.000 |
|  | Destination affective image |  |  |  | 0.236 | 2.602* | 0.011 |

Note.***: p＜.001, **: p＜0.01, *: p＜0.05

Table 4-16 Indirect effect decomposition table

|  | Effect value | Boot SE | Boot LLCI | Boot ULCI |
| --- | --- | --- | --- | --- |
| Total indirect effect | -0.066 | 0.071 | -0.219 | 0.063 |
| Ind1（Image presentation form of the name of scenic spot→Perceived advertising effectiveness→Visit intention） | -0.048 | 0.057 | -0.181 | 0.046 |
| Ind2（Image presentation form of the name of scenic spot→Perceived advertising effectiveness→Destination affective image→Visit intention） | -0.009 | 0.012 | -0.047 | 0.005 |
| Ind3（Image presentation form of the name of scenic spot→Destination affective image→Visit intention） | -0.009 | 0.027 | -0.084 | 0.035 |

Table 4-17 Chain mediation model of perceived advertising effectiveness and destination affective image

| Regression model | | Fit indicator | | | Coefficient significance | | |
| --- | --- | --- | --- | --- | --- | --- | --- |
| Outcome variable | Predictive variable | R | R^2^ | F | B | t | p |
| Visit intention |  | 0.257 | 0.066 | 7.349 |  |  |  |
|  | Text presentation form |  |  |  | 0.307 | 2.711** | 0.008 |
| Perceived advertising effectiveness |  | 0.241 | 0.058 | 6.407 |  |  |  |
|  | Text presentation form |  |  |  | 0.359 | 2.531* | 0.013 |
| Destination affective image |  | 0.386 | 0.149 | 8.990 |  |  |  |
|  | Text presentation form |  |  |  | -0.008 | -0.072 | 0.943 |
|  | Perceived advertising effectiveness |  |  |  | 0.298 | 4.132*** | 0.000 |
| Visit intention |  | 0.594 | 0.353 | 18.563 |  |  |  |
|  | Text presentation form |  |  |  | 0.162 | 1.654 | 0.101 |
|  | Perceived advertising effectiveness |  |  |  | 0.339 | 4.770*** | 0.000 |
|  | Destination affective image |  |  |  | 0.234 | 2.597* | 0.011 |

Note.***: p＜.001, **: p＜0.01, *: p＜0.05

Table 4-18 Indirect effect decomposition table

|  | Effect value | Boot SE | Boot LLCI | Boot ULCI |
| --- | --- | --- | --- | --- |
| Total indirect effect | 0.145 | 0.066 | 0.018 | 0.282 |
| Ind1（Text presentation of the scenic spot's name→Perceived advertising effectiveness→Visit intention） | 0.122 | 0.054 | 0.032 | 0.255 |
| Ind2（Text presentation of the scenic spot's name→Perceived advertising effectiveness→Destination affective image→Visit intention） | 0.025 | 0.019 | 0.003 | 0.089 |
| Ind3（Text presentation of the scenic spot's name→Destination affective image→Visit intention） | -0.002 | 0.028 | -0.076 | 0.043 |
